# Health-related quality of life and influencing factors in drug addicts based on the scale QLICD-DA: a cross-sectional study

**DOI:** 10.1186/s12955-022-02012-x

**Published:** 2022-07-14

**Authors:** Zilin Ma, Yuxi Liu, Chonghua Wan, Jianming Jiang, Xiaomei Li, Ying Zhang

**Affiliations:** 1 Key Laboratory for Quality of Life and Psychological Assessment and Intervention, Research Center for Quality of Life and Applied Psychology, School of Humanities and Management, Guangdong Medical University, Dongguan, 523808 China; 2 First People’s Hospital of Honghe Prefecture, Mengzi, 661100 China; 3 School of Public Health, Kunming Medical University, Kunming, 650500 China

**Keywords:** Health-related quality of life, Quality of life, Drug addicts, Influencing factor, QLICD-DA

## Abstract

**Background:**

Drug abuse has many negative effects not only on individuals but also on society. Nowadays, researchers pay a lot of attention to quality of life of drug addicts. However, there are few scales available to measure quality of life of drug addicts. The scale QLICD-DA (quality of life instrument for chronic diseases-drug addition) developed by modular approach could be used to measure quality of life of drug addicts with good validity, reliability and sensitivity.

**Objective:**

This study is aimed to understand the quality of life status and influencing factors in drug addicts by suitable sensitively scale, with the hypothesis of the quality of life in drug addicts being different from that of other peoples and possibly being influenced by many factors.

**Methods:**

By cluster random sampling method, 192 drug addicts at Kunming compulsory drug rehabilitation center were recruited to take part in the investigation. All participants completed the general information questionnaire and the scale QLICD-DA. We used a t-test to compare the scores of the quality of life of the participants with the norm (QOL scores from 1953 patients of 10 chronic diseases). A stepwise regression method was applied to explore the influencing factors of the quality of life in drug addicts.

**Results:**

192 participants ranged in age from 19 to 59 with an average age of 34.86. Most of them were male (70.3%), high school education level (67.7%) and of Han nationality (82.8%). The quality of life of drug addicts was lower than the norm in the physical domain, psychological domain, social domain, and general module, and the differences were statistically significant (*p* < 0.001). Sex and mode of drug abuse were the influencing factors in total score (*p* = 0.006) and specific module (*p* = 0.019). Past family atmosphere and the mode of drug abuse were the influencing factors in the general module (*p* = 0.027, *p* = 0.037).

**Conclusion:**

The quality of life of drug addicts was worse than that of patients with other chronic diseases, and the influencing factors of the quality of life of drug abusers were sex, mode of drug abuse, and past family atmosphere.

## Background

As everyone knows, the drug problem has always been present around the world, and it seriously affects human health. According to the World Drug Report 2020 of the United Nations, drug use is increasing all over the world. In 2018, it was estimated that there were 269 million drug addicts, accounting for 5.3% of the global population. Compared with 2009, there was an increase of 30% by 2018. With the development of the economy and society, the health problems of drug addicts have also attracted people’s attention, especially quality of life (QOL) or health-related quality life (HRQOL).

The concepts of QOL and HRQOL have been undergoing development in recent years. According to the WHO’s definition of quality of life, it is the individual's perception of their position in life in the culture and value system in which they live and it is associated with their goals, expectations, standards, and concerns[1]. It can be seen as a sense of well-being, incorporating psychological, physical, social, and mental aspects [2, 3]. Considering that many researchers have regarded drug addicts as patients with health problems [4, 5], this paper mentions QOL and HRQOL alternately.

Since the 1990s, in large-scale biomedical clinical trials, quality of life has always been at least a secondary endpoint [6, 7] for it is an important outcome measure in therapeutic research and service evaluation research [3]. Although the development and implementation of scales to measure the quality of life in different fields, such as cardiovascular disease, has become increasingly common, the researches on quality of life in drug addicts are still relatively scarce [8, 9]. Over the last 20 years, people have paid more attention to the quality of life of drug addicts, which have led many to realize that the problem of drug abuse is akin to having a chronic disease [4, 5].

However, most researchers use the general measures of QOL assessment such as the SF-36, SF-12, NHP, and WHOQOL-BREF. The problem is that these instruments are lacking sensitivity to assess the situation of drug dependence [3, 10]. As far as we know, in recent years, some specific drug dependency instruments have been developed, including injection drug use quality of life scale (IDUQOL) [10], drug user quality of life scale Spanish (IDUQOL- Spanish) [11], health-related quality of life for drug abusers test (HRQOLDA) [12], a quality of life instrument for patients with drug dependence (QOL-DA) [3] and the drug user quality of life scale (DUQOL-22 items) in Australia [13].

IDUQOL is designed to capture the unique and individual circumstances that determine the quality of life of injection drug users. It has the advantages of good content validity, but it only focuses on the quality of life of injecting drug users, and the completion time is relatively long, which is not conducive to the actual situation [10]. The DUQOL-Spanish is based on the IDUQOL. But it differs from the IDUQOL in that it includes a new domain of life: “sense of future”. It is revised based on IDUQOL and can be used for injection drug users or non-injection drug users. It is easy to complete and accepted by most people. However, the questionnaire is not compared with another QOL assessment tool, and it is not known whether it can effectively measure the quality of life of drug users [13]. The HRQOLDA is a 20-item Likert type instrument that has been validated in Spain to assess QOL amongst drug abusers and DUQOL-22 items in Australia is the first time to be tested in a sample of individuals seeking treatment. The latter two tools are both relatively easy to operate, with the time for completion being relatively short, and being more likely accepted by participants [12, 13]. However, they need further validation in practice [3, 13].

Besides, a self-administered quality of life scale for patients with drug addicts (QOL-DA) was developed by Wan et al. in 1997 [3]. It has better validity, reliability, especially responsiveness than generic instruments such as SF-36 since it captures the specific issues related to drug addiction.

However, all of these specific instruments are not developed by modular approach. Considering same-class diseases often share many things in common, a popular modular approach in recent years has been used to develop a general module for a class of diseases and then additional modules to capture individual differences in different people and diseases. To meet this trend, we have developed the Chinese QOL instruments system called QLICD (Quality of Life Instruments for Chronic Diseases) by combining a general module and disease-specific modules. The updated second edition of this system includes a general module (QLICD-GM) and 34 specific modules for 34 different diseases. The QLICD-DA V2.0 [14, 15] is a specific instrument by this modular approach with the QLICD-GM [16] being used for all chronic diseases and the specific module only for drug additions. The QLICD-DA (V2.0) has been proved to have good reliability and validity [14, 15]. It can be used to measure the quality of life of drug addicts sensitively, which overcomes the deficiency of the universal scale in measuring the quality of life of drug addicts (insensitivity). At the same time, it can be used to compare the quality of life of different chronic disease patients with the general module, which makes up for the lack of systematic and consistent of the previous development of drug addicts’ specific scale. Therefore, this paper is aimed to use the QLICD-DA to explore the quality of life and the influencing factors of drug addicts, and to provide a reference for health policymakers. It is anticipated that the results can be used for making decisions in clinical trials as well as for individual management of this disease.

## Methods

### Study design and setting

A Cross-sectional Study was carried out to the participants who meet the requirements from the Kunming Compulsory Drug Rehabilitation Center during the period of survey. The center is located in outskirts of the Kunming city. It is a specialized institution focusing on mandatory detoxification and rehabilitation of drug addicts.

Two questionnaires of the QLICD-DA and the general information including sex, age, and education level etc. were used to investigate the participants.

### Participants

By cluster random sampling method, all drug addicts who meet the requirements at this center during the period of survey were recruited to take part in the investigation. The inclusion criteria comprised: (a) participants are heroin dependent patients in accord with the DSM-IV criteria for substance abuse disorders (substance dependence and abuse disorders); (b) have a certain level of reading and writing skills, and can understand the content of the questionnaire; (c) volunteer to participate in the assessment. The exclusion criteria comprised: (a) illiterate; (b) unconscious and unable to clearly express their inner feelings; (c) serious diseases.

### Instruments and variables

#### The general information questionnaire

The general information questionnaire is a self-made questionnaire, including questions about demographic and sociological characteristics (age, gender, national origin, marital status, medical insurance, occupation, education, income) and the condition of drug abuse (the economic status of the residence, past family atmosphere, the social atmosphere of the residence, lifestyle, nutritional status, the greatest drug desire after detoxification, length of drug abuse, times of abstaining from the drug, mode of drug abuse, and typical drug dosage). In this research, family atmosphere and social atmosphere are the ways how family members or closers get along with each other, which are perceived and evaluated by themselves on five levels from very bad to excellent (1 not at all, 2 a little bit, 3 somewhat, 4 quite a bit, 5 very much). Also, nutritional status is the state and conditions of nutrition perceived and evaluated by themselves on five levels from very bad to excellent (1 not at all, 2 a little bit, 3 somewhat, 4 quite a bit, 5 very much).

#### The scale QLICD-DA

The QLICD-DA (V2.0) is composed of the general module QLICD-GM (V2.0) and the drug addiction specific module for it was developed by modular approach. The general module QLICD-GM (V2.0) includes 3 domains and 28 items (9 items for physical function, 11 items for psychological function, and 8 items for social function) based on the WHO’s definition and framework of the quality of life, while the specific module has 16 items regards to withdrawal symptoms and side effects.

The score of the QLICD-DA can be divided into two categories: (1) raw score, and (2) standardized score, similar to other QLICD scales such as QLICD-PT for tuberculosis [17]. After investigation, the raw scores of items, domains and overall scale were calculated. The answer options for each item of the QLICD-DA are quantified using a 5-point Likert scale (1-not at all, 2-a little bit, 3-somewhat, 4-quite a bit, 5-very much). Therefore, the positively stated items which have better quality of life with higher answer options, directly obtain scores from 1 to 5 points, while the negatively stated items are reversed. The sum of the scores of each item in the same domain constituted the raw score of the domain. The score of the general module is calculated by summing the score of the physical domain, psychological domain, and social domain. The general module and the specific module score constitute the raw score of the overall scale.

For comparison, all domains/modules and the overall scores were linearly converted to a 0–100 scale using the formula: SS = (RS − Min) × 100/R, where SS, RS, Min, and R represent the standardized score, raw score, minimum score, and range of scores, respectively.

### Survey methods

The study population was limited to heroin dependent patients at any stages who were able to read and understand the questionnaires. By cluster random sampling method, all heroin dependent patients who meet the requirements at this center during the period of survey were study participants. On the first day of their enrollment into the center, the investigators (a well-trained medical student) explained the survey and the questionnaires to them and obtained informed consent from those who agreed to participate in the study and met the inclusion criteria. These participants were asked to fill out the general information questionnaire and the QLICD-DA during their interview with an investigator. Answers were checked immediately each time by the investigators in order to ensure its integrality. If missing values were found, the questionnaires would be returned to the patients to fill in the missing item.

The survey continued to meet sample size at least 165 cases. The sample size was computed by following statistical formula with ${\rm Z}_{\alpha /2} = 1.96$, allowable error δ = 2:

$$n = \left( {\frac{{z_{{a/2}} \sigma }}{\delta }} \right)^{2}$$

Here n is estimated sample size, and σ is standard deviation of the QOL score of drug addicts. In this paper, we used the standard deviation (σ = 13.1) of another scale also ranging from 0 to 100 to estimate it because no QOL data of drug addicts measuring by QLICD-DA.

### Data analysis

The descriptive statistics were used to analyze the total score and domain scores of quality of life in drug addicts and obtained the extremum and the average. Then the t-test was used to compare the quality of life in drug addicts with the norm (the QOL scores from 1953 patients of 10 chronic diseases). One-way analysis of variance (ANOVA) was used to screen the independent variables that possibly affect the quality of life of the participants. Stepwise multiple linear regression analyses (backward selection method) were performed with the total score and the score of each domain being as the dependent variables, respectively (*p*-value in=0.05 and *p*-value out = 0.10). The categorical independent variables were recoded (assignment) before stepwise linear regression analysis (see Table 1 in detail).

**Table 1:** The variable assignment of multiple linear regression

Variables	Description/recoding
Age (X1)	Measured value
Sex (X2)	1 Male, 2 Female
Nation (X3)	1 Han, 2 Others
Education (X4)	1 Primary school, 2 Junior middle school, 3 High school, 4 Junior college, 5 College or higher
Income (X5) Length of drug abuse (X6) Times abstaining from drug (X7)	Measured value Measured value Measured value
Drug dependence (X8)	1 not at all, 2 a little bit, 3 somewhat, 4 quite a bit, 5 very much
Mode of drug abuse(X9) Drug dosage (X10) Economic status (X11) Family atmosphere (X12) Social atmosphere (X13) Life style (X14) The greatest desire after detoxification (X15)	1 Intravenous injection, 2 Others Measured value 1 not at all, 2 a little bit, 3 somewhat, 4 quite a bit, 5 very much 1 not at all, 2 a little bit, 3 somewhat, 4 quite a bit, 5 very much 1 not at all, 2 a little bit, 3 somewhat, 4 quite a bit, 5 very much 1 Live alone, 2 Others 1 Complete independence, 2 Others

## Results

There were 192 participants, ranging in age from 19 to 59, with an average age of 34.86 ± 0.59. Most of them were male (*n* = 135, 70.3%) and of Han nationality (*n* = 159, 82.8%). In terms of personal income, more than a third of the participants’ incomes were less than 1,000 yuan/year (*n* = 45, 34.9%). Besides, more than half of them were single or divorced (*n* = 135, 70.3%). In regard to education level, 60 people have a primary school education(accounting for 31.3%), 93 people have junior high school education(accounting for 48.4%), 37 people have senior high school (accounting for 19.3%), and 2 people have a college degree or above(accounting for 1%). See Table 2 in detail.

**Table 2:** Socio-demographic characteristics of the Sample (*n* = 192)

Characteristics	*N*	%
*Gender*		
Male	135	70.3
Female	57	29.7
*Nation*		
Han	159	82.8
Others	33	17.2
*Age*		
< 30	49	25.5
30–39	82	42.7
> 39	61	31.8
*Income*		
0–1000	45	34.9
1001–5000	15	7.8
5001–10,000	22	17.1
*Marital status*		
Married	57	29.7
Others	135	70.3
*Medical insurance*		
Self-paid	140	72.9
Public insurance	39	15.1
Others	13	6.8
*Occupation*		
Worker	26	13.5
Farmer	71	37.0
Self-employed	40	20.8
Others	55	28.6
*Education*		
Primary school	60	31.3
Junior high school	93	48.4
Senior high school	37	19.3
College or higher	2	1

Table 3 showed the participants’ quality of life in six aspects, which were the physical domain, psychological domain, social domain, the specific module, the general module, and the total score. We found that the average physical score was higher compared with the other domains (*p* < 0.05) with mean = 58.67 ± 9.74. Besides, one notable finding was that the average score in the specific module was the lowest (mean = 36.84 ± 19.24) and the range was the widest with a difference of 95.31 points (max = 100.00, min = 4.69). Compared with the other two domains (physical domain and social domain), the average score of the psychological domain was lower. Compared with the norm, we found that the average score of the participants was dramatically lower than the norm and this difference was statistically significant (*p* < 0.05). We also found that the average score of the psychological domain was also dramatically lower than the other two domains (physical domain and social domain). The average score of the participants in each domain was lower than the norm and the differences were statistically significant (*p* < 0.05).

**Table 3:** Quality of life scores in drug addicts based on the QLICD-DA and comparisons with the norm

Domains	Maximum	Minimum	Drug addicts (mean ± SD)	Norm (mean ± SD)	t	*p*
Physical domain (PHD)	94.44	30.56	58.67 ± 9.74	62.45 ± 15.37	− 5.383	< 0.001
Psychological domain (PSD)	84.09	15.91	50.57 ± 14.47	62.23 ± 17.79	− 11.170	< 0.001
Social domain (SOD)	87.50	18.75	57.18 ± 14.51	72.54 ± 15.55	− 14.670	< 0.001
The general module (CGD)	79.46	26.79	55.06 ± 9.84	78.24 ± 43.91	− 32.629	< 0.001
Total (TOT)	81.82	25.00	48.43 ± 10.02	–	–	–
The Specific module (SPD)	100.00	4.69	36.84 ± 19.24	–	–	–

The relevant scores from 1953 patients of 10 chronic diseases including hypertension, diabetes, etc. were considered as the norm

By stepwise linear regressions, we found that there were no factors can be selected into the model with significant differences for three domains of the physical, psychological and social. We found that the sex and mode of drug abuse were the major influencing factors of the total score and the specific module. Besides, the general module's significant influencing factors were past family atmosphere and mode of drug abuse. Overall, sex, mode of drug abuse, and past family atmosphere were the main influencing factors of the quality of life of the participants, See Table 4 in detail.

**Table 4:** Impact factors on quality of life in drug addicts selected by stepwise linear regressions

Domains	Factors	B	Std. Error	Standardized B	t	*p*
The total (TOT)	Constant	61.831	3.270		18.910	0.000
	Mode of drug abuse	− 5.077	1.800	− 0.244	− 2.820	0.006
	Sex	− 4.744	1.952	− 0.210	− 2.431	0.000
The general module (CGD)	Constant	51.888	4.384		11.835	0.000
	Past family atmosphere	2.457	1.095	0.195	2.244	0.027
	Mode of drug abuse	− 3.671	1.741	− 0.183	− 2.108	0.037
The Specific module (SPD)	Constants	77.459	8.298		9.334	0.000
	Sex	− 14.910	3.647	− 0.349	− 4.088	0.000
	Mode of drug abuse	− 7.809	3.283	− 0.198	− 2.379	0.019

## Discussions

In this research, we used the QLICD-DA scale to measure the quality of life of drug addicts. Compared with generic QOL instruments, the QLICD-DA scale has better validity, reliability, and responsiveness. This scale adopts a combination of a general module and the specific module, which is suitable for the Chinese culture and is sensitive to the factors affecting the quality of life of drug-dependent people. The QLICD-DA has been applied by many Chinese scholars, reflecting its stability and effectiveness. For example, Zhang et al. [18] used the QLICD-DA scale to measure the quality of life and its influencing factors for maintenance patients and Guo et al. [19] also measured the quality of life and its influencing factors on male drug-dependent people in compulsory treatment. Therefore, we can use the QLICD-DA to identify the factors influencing the quality of life in drug addicts more directly and effectively.

As previous studies have suggested that drug addiction is a chronic disease [4, 5], the quality of life scores measured by QLICD-GM from 1953 patients of 10 chronic diseases including hypertension, coronary heart disease, COPD, diabetes, rheumatoid arthritis etc., were considered as the norm of chronic diseases for large samples. Therefore, the score of the patients with drug addiction was compared with the norm of chronic diseases in three general aspects including physical domain, psychological domain and social domain. The results showed that the quality of life in drug addicts is lower than that for patients with other chronic diseases (the norm). A similar observation has been made by Smith and Larson [20]. Susannah et al. [21] considered that heroin dependence is a serious chronic disease, which has harmful effects on physical and mental health. Compared with patients with other chronic diseases, the decline in the quality of life in drug addicts is determined by the comprehensive negative impact of drug use in several areas. Moreover, drug addicts usually only seek treatment when they suffer from serious consequences, while patients with other chronic diseases pay more attention to their diseases and will engage in a certain degree of prevention and health care consumption [22]. Furthermore, drug addicts are more likely to experience criminal charges, which put them under various pressures.

The psychological status of drug addicts is relatively weak. Mònica Astals et al. [23] also found that the psychological function of heroin patients was seriously damaged, which is similar to the findings of our study. Anxiety disorders and mood disorders are common mental disorders among people who abuse drugs [24]. There are some possible reasons for this. Firstly, drug abuse easily leads to adverse consequences, and may even prompt the performance of criminal acts, which applies a psychological burden. What's more, the feeling of withdrawal when attempting to stop drug abuse is strong and unbearable, causing physical and mental suffering. Second, substance abusers are not respected or accepted in society, causing them to lose the support of their friends and family. There is no doubt that these factors together will generate many negative emotions. Therefore, the mental health of drug addicts must be taken seriously.

Our findings also found that sex played an important role in the total score and the specific module. The quality of life of female drug abusers is worse than that of male drug abusers, which was consistent with Karow et al. [25]. There are some possible reasons for this. First, although the number of women who abuse drugs is relatively low, their dependence tends to be more severe than that of men. What’s more, Chinese society gives men more personal freedom and it has a lot of restrictions on women. The social tolerance of female drug users is much lower than that of male drug users, so female drug users are more likely to be rejected by their relatives and friends [26]. Social stigma would increase these women’s sense of helplessness and negative emotions. Also, drug use increases women’s risk of AIDS/HIV, which is possibly due to needle sharing or prostitution [27]. In the present study, the female drug abusers were more likely to suffer from overt mental illness than male drug abusers, such as depression [28].

Besides, our findings indicated that the modes of drug abuse are also an important influencing factor, which was found to be a common factor affecting the total score, general module, and the specific module. The quality of life of people who use other modes of drug use is lower than those who inject drugs. It was conjectured that although intravenous injection is harmful to the human body, other ways of administering drugs such as by inhalation, will lead to stronger drug addiction. What’s more, inhalation users are more likely to relapse after treatment.

At the same time, the past family atmosphere cannot be ignored, with bad past family atmosphere worsening their quality of life. Al-Kandari et al. [29] reported that one person in the family taking drugs often leads to another, especially in situations where a parent abusing drugs leads to the children doing so as well. An uneasy and tense family atmosphere can make children feel insecure. Consequently, they tend to rely on drugs to compensate for their anxiety.

### Limitations

The participants in this study were only selected from one detoxification center, and only limit to heroin dependent patients, which may affect the generalizability of the study. Additional community-based studies with larger sample sizes are needed. In addition, this study only focuses on some socio-demographic factors and the conditions of drug abuse. The factors on other environments and among other types of drug abuses need to be further explored. Future studies should investigate why quality of life is linked to socio-demographic factors, and also factors on status of drug abuse.

## Conclusions

It concluded that the quality of life in drug addicts was worse than that of patients with other chronic diseases, and the influencing factors of the quality of life of drug abusers were sex, mode of drug abuse, and past family atmosphere. It should be pay more attention to the quality of life of drug addicts, and effective measures should be taken in order to improve their quality of life.

## Data Availability

The data (two formats: SPSS and Excel) can be available by request from Prof. Chonghua Wan (Email: wanchh.hotmail.com).
